# Supplementary material for: Developmental self-reactivity determines pathogenic Tc17 differentiation potential of naive CD8+ T cells in murine models of inflammation
Source: Nat Commun. 2024 Apr 4;15:2919. doi: 10.1038/s41467-024-47144-4 (PMC10994929; doi:10.1038/s41467-024-47144-4)
Supplement: Supplementary file 1 — Supplementary Information [file 41467_2024_47144_MOESM1_ESM.pdf]

Supplementary Information

Developmental self-reactivity determines pathogenic Tc17 differentiation potential of naive CD8$^+$ T cells under inflammatory conditions

Gil-Woo Lee, Young Ju Kim, Sung-Woo Lee, Hee-Ok Kim, Daeun Kim, Jiyoung Kim, You-Me Kim, Keunsoo Kang, Joon Haeng Rhee, Ik Joo Chung, Woo Kyun Bae, In-Jae Oh, Deok Hwan Yang, and Jae-Ho Cho

Supplementary information includes:

Supplementary Figures 1−7

Supplementary Figure legends 1−7

Supplementary Tables 1 and 2

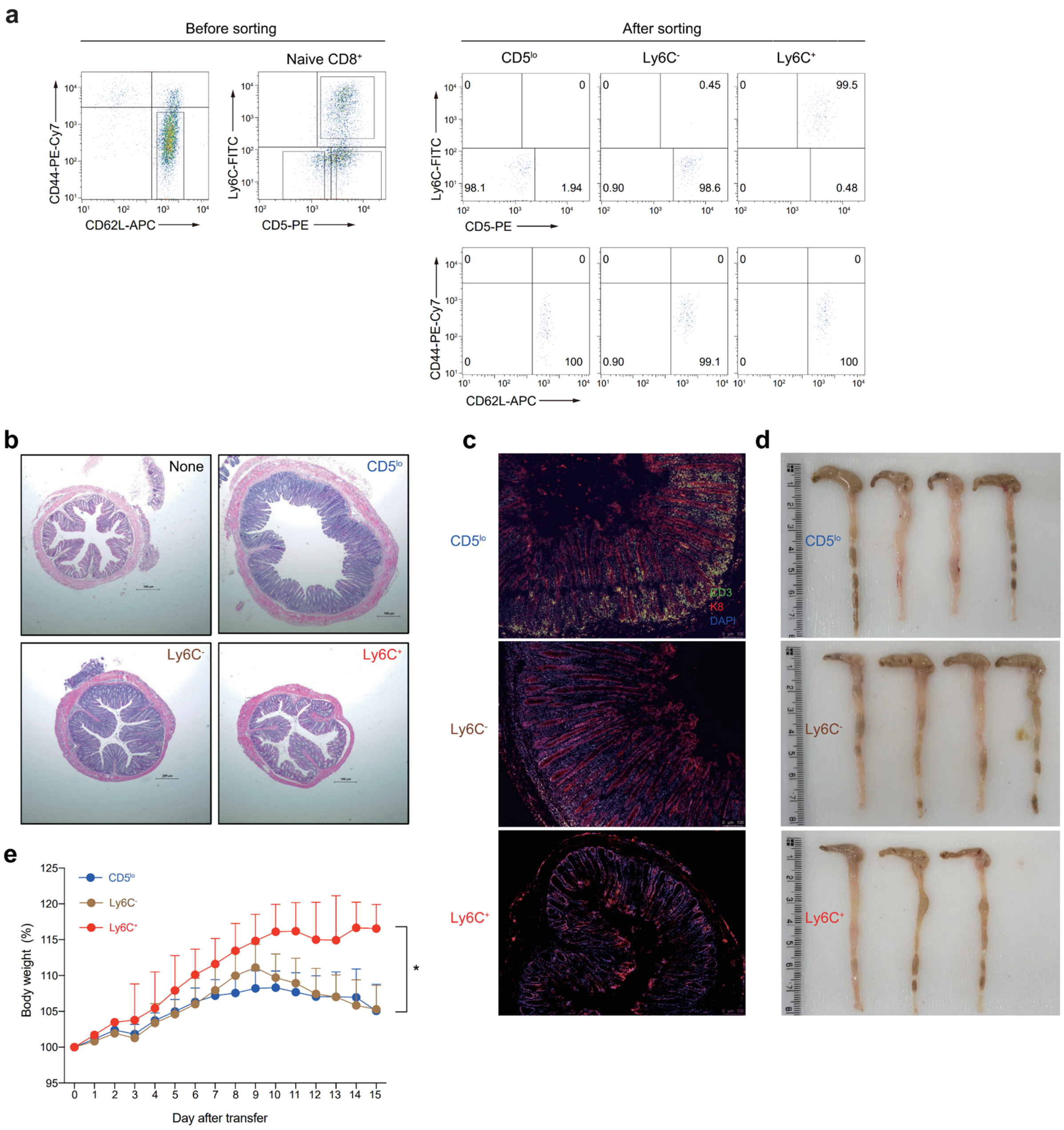

Supplementary Fig. 1

Pathologic symptoms induced by different CD8$^+$ T$_N$ subsets in *Rag1*$^{-/-}$ mice.

a, Sorting purity of naive CD8$^+$ T cells subsets. **b,** Representative H & E staining images (magnification, 40×). **c,** Representative immunofluorescence images (magnification, 200×). **d,** Representative photo images for day 14 LI. **e,** Body weight changes of *Rag1*$^{-/-}$ recipients after adoptive transfer with CD8$^+$ T$_N$ subsets (n=6). Data is representative of three independent

experiments and presented as the mean ± SD (**d**). Statistical significance by Mann-Whitney test. *, $P < 0.05$; **, $P < 0.01$; ***, $P < 0.001$; ****, $P < 0.0001$. Source data and exact P value are provided as a Source Data file.

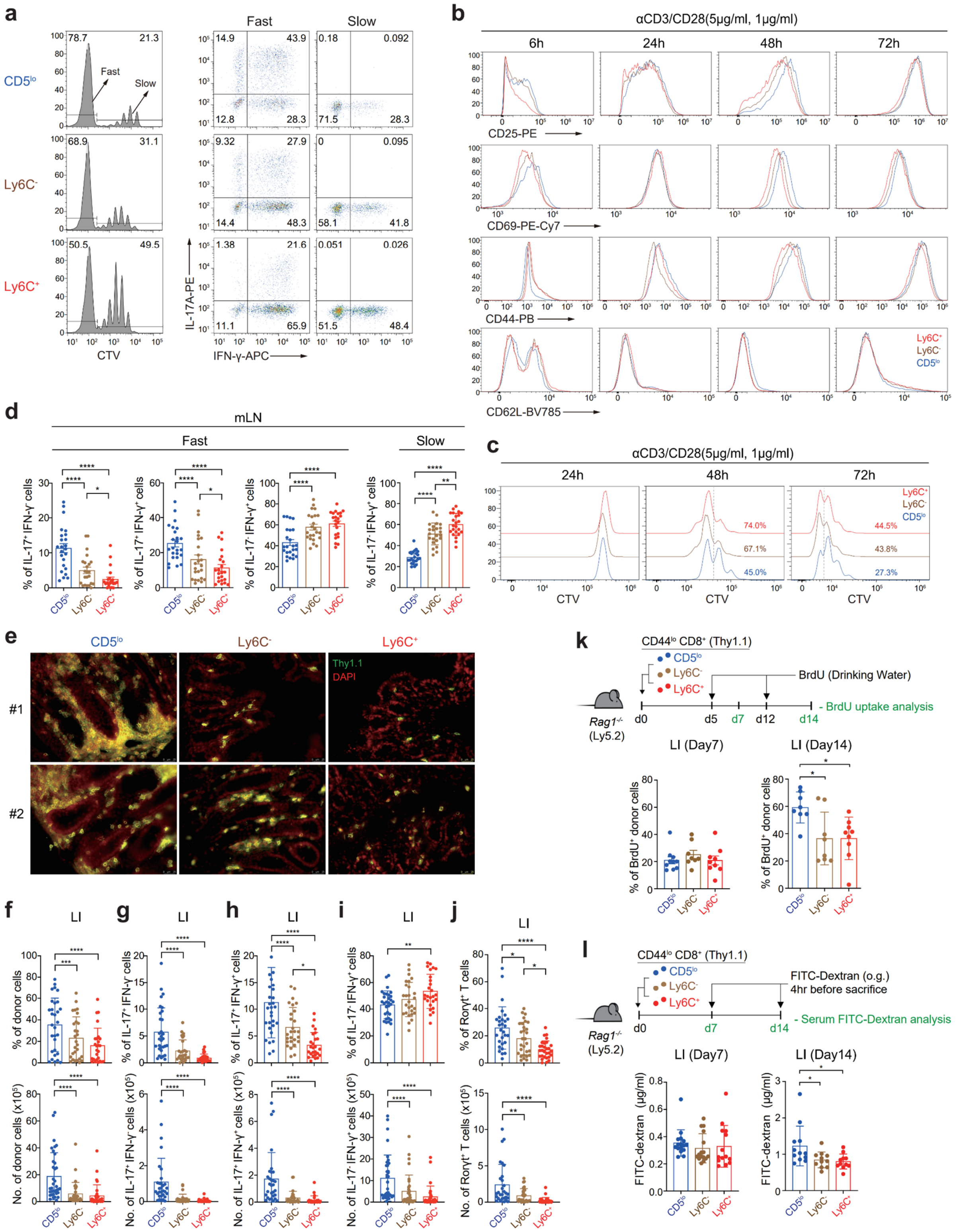

Supplementary Fig. 2

Proliferation, colonic infiltration, and IL-17/IFN-γ production of adoptively transferred $CD8^+$ T_N subsets in *Rag1*$^{-/-}$ mice.

a, CTV-labeled CD5^{lo}, Ly6C^{-}, and Ly6C^{+} CD8^{+} T_N subsets were adoptively transferred into *Rag1*$^{-/-}$ recipients and the proliferation of donor cells were analyzed at day 7 by measuring CTV dilution. Representative histograms (left) and FACS plots (right) show CTV dilution and IL-17A/IFN-γ production, respectively, for fast and slow proliferative donor cells. **b,** CD5^{lo}, Ly6C^{-}, and Ly6C^{+} CD8^{+} T_N subsets were stimulated with plate-bound anti-CD3 and anti-CD28, and analyzed for expression patterns of various activation markers (CD25, CD69, CD44, and CD62L) at different time points. **c,** T_N subsets were labeled with CTV and then cultured on plates coated with anti-CD3 and anti-CD28. Cell proliferation was analyzed by flow cytometry at 24, 48, and 72 h after TCR stimulation. **d,** Percentage of IL-17A^{+}IFN-γ^{-}, IL-17A^{+}IFN-γ^{+}, and IL-17A^{-}IFN-γ^{+} cells for fast (left) and slow (right) proliferative donor subsets in day 7 mLN (CD5^{lo} n=24, Ly6C^{-} n=23 and Ly6C^{+} n=22). **e–j,** Immunofluorescence images for Thy1.1^{+} donor cells **(e;** scale bar, 25 μm), the percentage and number of total donor (**f,** CD5^{lo} n=30, Ly6C^{-} n=28 and Ly6C^{+} n=26**)**, IL-17A^{+}IFN-γ^{-} **(g,** CD5^{lo} n=30, Ly6C^{-} n=28 and Ly6C^{+} n=26**)**, IL-17A^{+}IFN-γ^{+} (**h,** CD5^{lo} n=30, Ly6C^{-} n=28 and Ly6C^{+} n=26**)** IL-17A^{-}IFN-γ^{+} (**i,** CD5^{lo} n=30, Ly6C^{-} n=28 and Ly6C^{+} n=26), and Rorgt^{+} **(j,** n=29**)** cells from day 14 LI. **k.** Percentage of BrdU^{+} cells in donor cells from LI (left; CD5^{lo} n=10, Ly6C^{-} n=8 and Ly6C^{+} n=9, right; CD5^{lo} n=8, Ly6C^{-} n=8 and Ly6C^{+} n=9). **l.** *In vivo* gut permeability assay from mice receiving individual CD8^{+} T_N subsets (left; CD5^{lo} n=16, Ly6C^{-} n=16 and Ly6C^{+} n=14, right; CD5^{lo} n=12, Ly6C^{-} n=11 and Ly6C^{+} n=11). Data are pooled from three **(d)**, two independent (**k,l**), and four **(f–i,j)** independent experiments and presented as the mean ± SEM (**d-j**). Statistical significance by two-way ANOVA Multiple comparisons. *, $P < 0.05$; **, $P < 0.01$; ***, $P < 0.001$; ****, $P <$ 0.0001. Source data and exact *P* value are provided as a Source Data file.

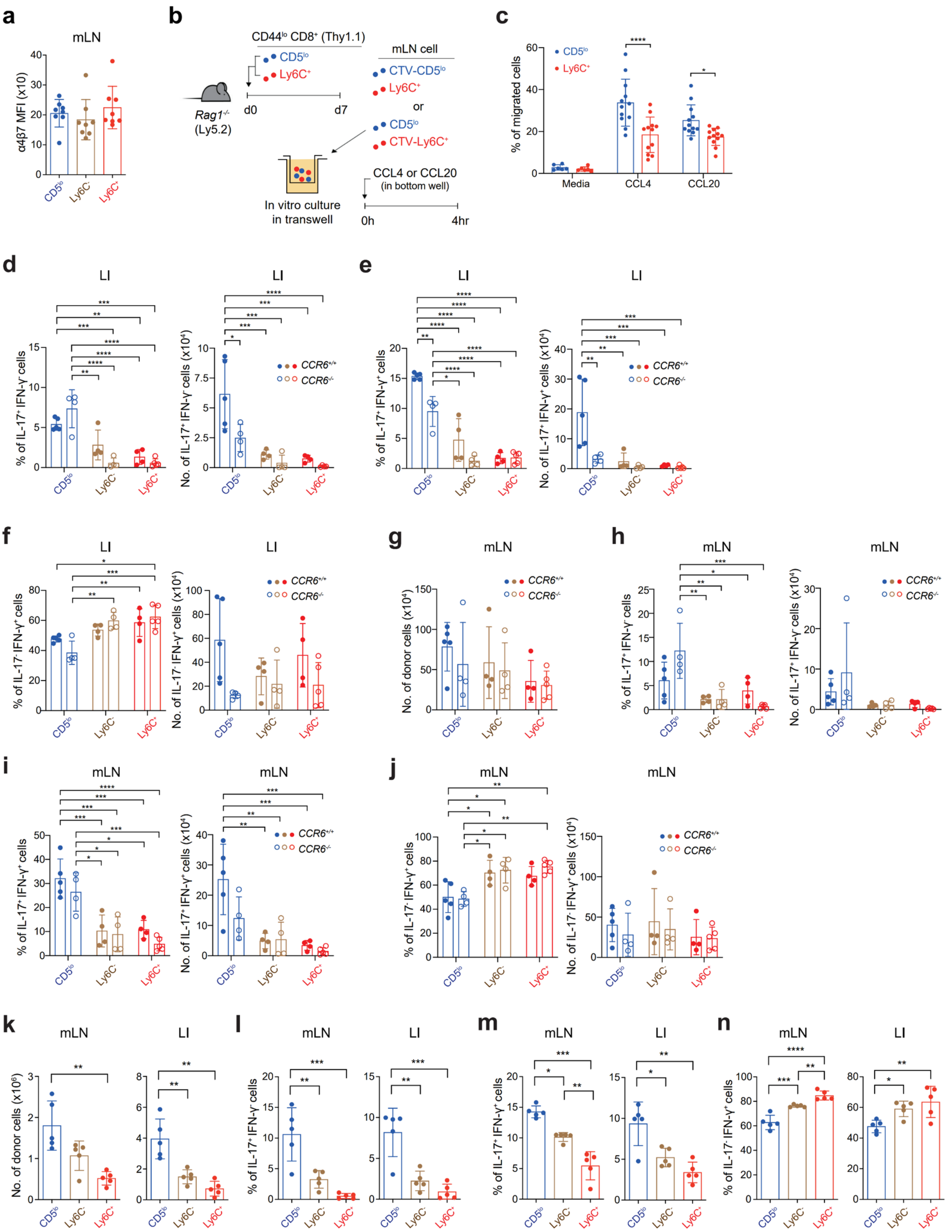

Supplementary Fig. 3

The impact of CCR6 deficiency on IL-17/IFN-γ production of adoptively transferred CD8⁺ T_N subsets in *Rag1*$^{-/-}$ mice.

a, Expression levels (as the mean fluorescence intensity, MFI) of α4β7 of transferred donor subsets from day 7 mLN **(**n=8**)**. **b,** Experimental design for **c**. **c,** Chemotactic migration was analyzed for transferred $CD5^{lo}$ and $Ly6C^{+}$ cells collected from day 7 mLN of *Rag1*$^{-/-}$ recipients **(**Media; n=6, CCL4 and CCL20; n=12**)**. Data were pooled from two independent experiments **(c,** n=3–6 mice/experiment**)**. **d–j,** Percentage and number of IL-17A^{+}IFN-γ^{-} (**d,h),** IL-17A^{+}IFN-γ^{+} **(e,i)**, and IL-17A^{-}IFN-γ^{+} **(f,j)** cells, were analyzed at day 14 after adoptive transfer with either *Ccr6*$^{+/+}$ or *Ccr6*$^{-/-}$ $CD8^{+}$ T_N subsets. **k–n,** Number of donor cells **(k)** and the percentage of IL-17A^{+}IFN-γ^{-} **(l)**, IL-17A^{+}IFN-γ^{+} **(m)**, and IL-17A^{-}IFN-γ^{+} **(n)** cells analyzed in mLN and LI at day 21 after transfer with *Ccr6*$^{-/-}$ $CD8^{+}$ T_N subsets **(d–j,** n=4–5; **k–n,** n=5**)**. Data are representative of two independent experiments and presented as the mean ± SD **(a–l)**. Statistical significance by two-way ANOVA Multiple comparisons. *, $P < 0.05$; **, $P < 0.01$; ***, $P < 0.001$; ****, $P < 0.0001$. Source data and exact *P* value are provided as a Source Data file.

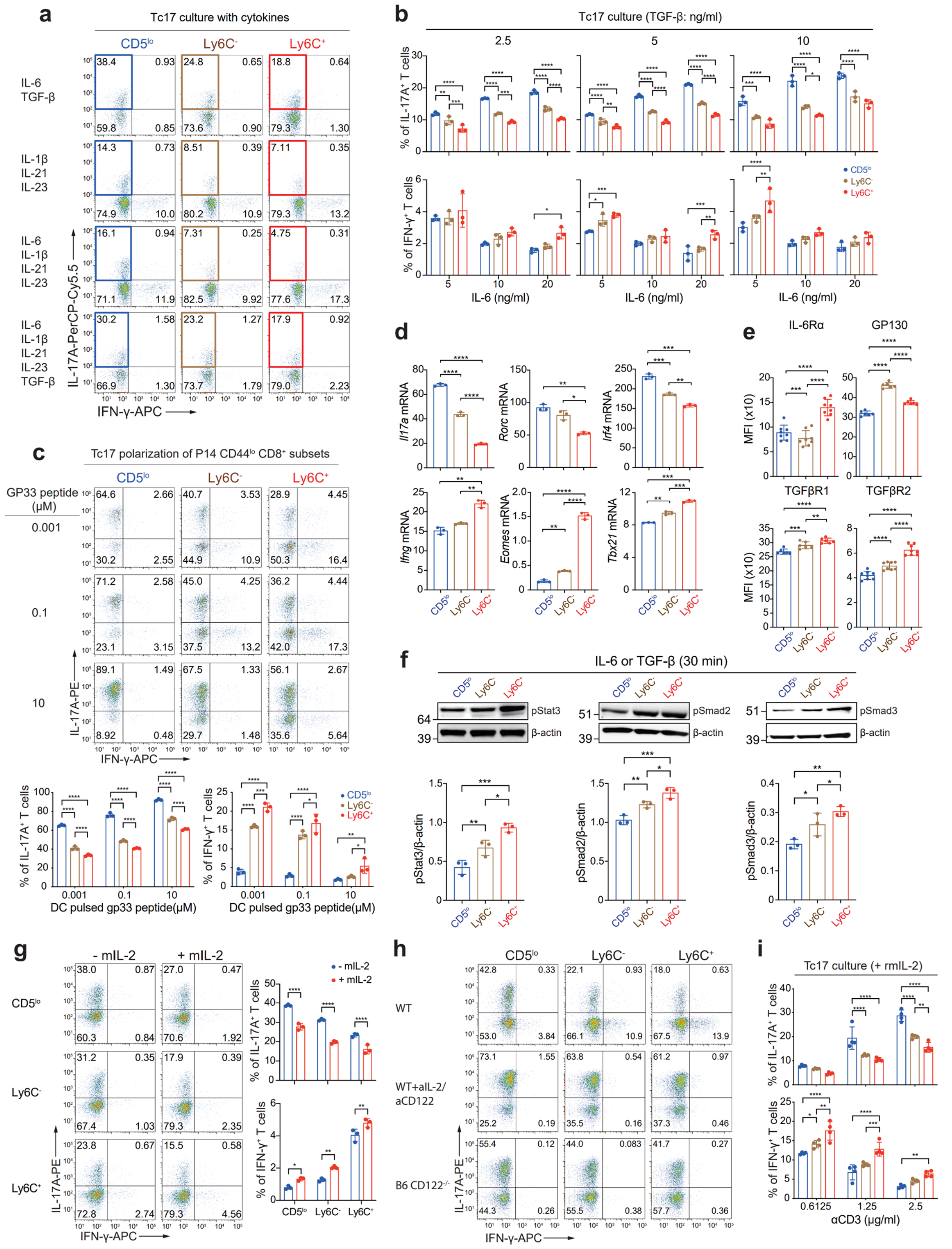

Supplementary Fig. 4

Differential Tc17-skewing potential of CD8+ TN subsets under various Tc17-polarizing conditions *in vitro*.

a, Representative FACS plots for IL-17A/IFN-γ production after *in vitro* culture with B6 $CD8^+$ T_N subsets under various Tc17-polarizing conditions. **b,** Percentage of IL-17A$^+$ and IFN-γ$^+$ cells after Tc17-polarizing culture with B6 $CD8^+$ T_N subsets in the presence of various concentrations of IL-6 and TGF-β **(**n=3 per experiment**)**. **c,** Representative FACS plots for IL-17A/IFN-γ production from P14 $CD8^+$ T_N subsets stimulated with GP33 peptide-pulsed dendritic cells under Tc17-polarizing condition. **d,** qRT-PCR data for *Il17a, Rorc,, Irf4, Ifng, Eomes,* and *Tbx21* after Tc17-polarizing culture with B6 $CD8^+$ T_N subsets **(**n=3 per experiment**)**. **e,** Expression levels of IL-6Rα, GP130, TGFβRI, and TGFβRII on B6 $CD8^+$ T_N subsets **(**n=6–8 mice/group**)**. **f,** Levels of p-STAT3, p-SMAD2, and p-SMAD3 (shown in representative blot images, top, and intensity relative to β-actin, bottom) **(**n=3 per experiment**)**. **g,** Representative FACS plots for IL-17A/IFN-γ production (left) and the percentage of IL-17A$^+$ and IFN- γ$^+$ cells (right) after Tc17-polarizing culture with B6 $CD8^+$ T_N subsets in the presence or absence of rmIL-2 **(**n=3 per experiment**)**. **h,** Representative FACS plots for IL-17A/IFN-γ production after Tc17-polarizing culture with B6 $CD8^+$ T_N subsets in the presence (middle) or absence of anti-IL-2/CD122 (top) or with *Cd122*$^{-/-}$ $CD8^+$ T_N subsets (bottom). **i,** Percentage of IL-17A$^+$ and IFN-γ$^+$ cells after Tc17-polarizing culture of B6 $CD8^+$ T_N subsets with various concentrations of anti-CD3 and rmIL-2 **(**n=3 per experiment**)**. Data is representative of two (**a–d,e**) to three independent experiments (**f,g,i**) and presented as the mean ± SD **(b–g,i)**. Statistical significance by two-way ANOVA Multiple comparisons. *, $P < 0.05$; **, $P < 0.01$; ***, $P < 0.001$; ****, $P < 0.0001$. Source data and exact *P* value are provided as a Source Data file.

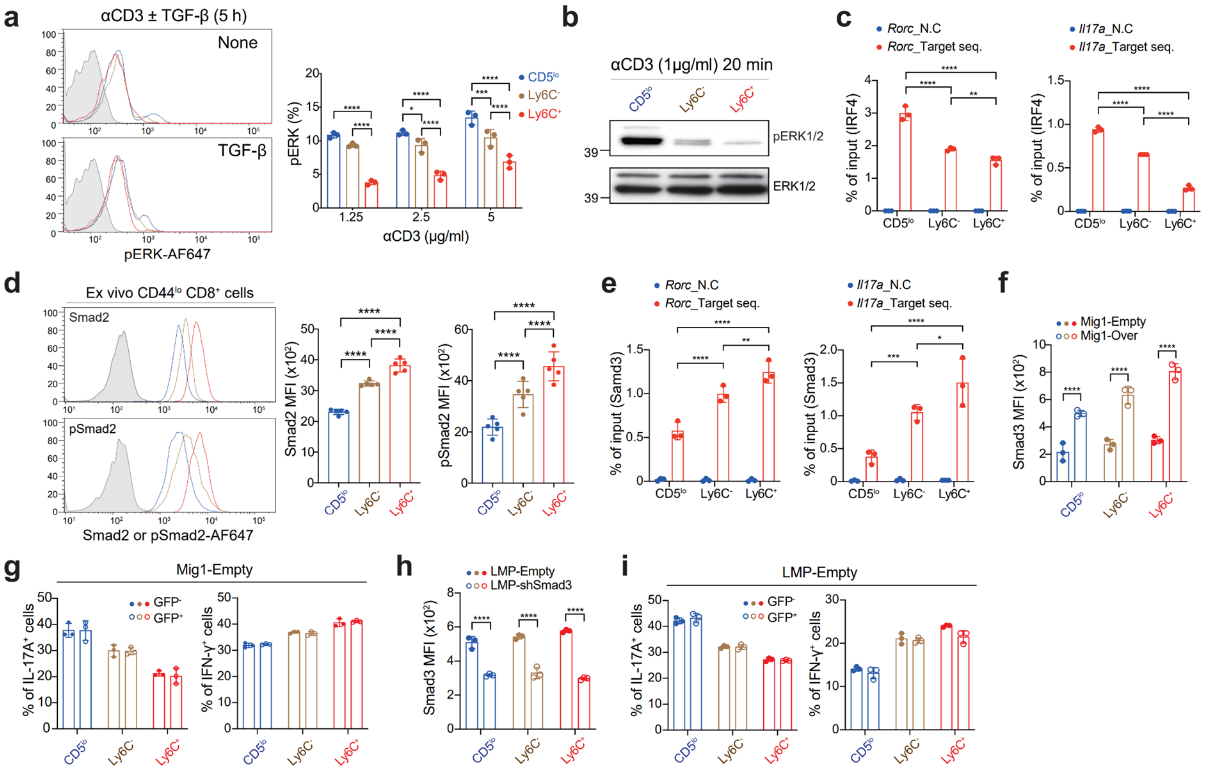

Supplementary Fig. 5

Differential levels of TCR-induced ERK activation and endogenous SMAD2 expression in CD8^{+} T$_{N}$ subsets.

a, Histograms (left) and percentages (right) for p-ERK expression among CD8^{+} T$_{N}$ subsets stimulated with either anti-CD3 in the presence or absence of TGF-β (left) or with various concentrations of anti-CD3 (right) **(**n=3 per experiment**). b,** Representative blot images of p-ERK after 20 min stimulation with anti-CD3. **c,** B6 CD8^{+} T$_{N}$ subsets were activated under Tc17-polarizing conditions for 72 h and subjected to ChIP using IRF4 antibody. Eluted DNA was analyzed by qPCR **(**n=3 per experiment**). d,** Endogenous levels of SMAD2 and p-SMAD2 in *ex vivo* B6 CD8^{+} T$_{N}$ subsets shown in histogram (left) and MFI (right) **(**n=5 mice/group**). e,** B6 CD8^{+} T$_{N}$ subsets were activated under Tc17-polarizing conditions for 72 h and subjected to ChIP using SMAD3 antibody. Eluted DNA was analyzed by qPCR **(**n=3 per experiment**). f,** Expression levels of SMAD3 from Tc17-polarized CD8^{+} T$_{N}$ subsets transduced with either MigR-1 control (empty) or MigR-1 encoding *SMAD3* (over) **(**n=3 per experiment**). g,**

Percentage of IL-17A$^+$ and IFN-γ$^+$ cells in GFP$^-$ and GFP$^+$ cells transduced with MigR-1 empty vector control (n=3 per experiment). **h,** Expression levels of SMAD3 for Tc17-polarized CD8$^+$ T_N subsets transduced with LMP empty vector control or LMP vector containing *SMAD3* shRNA. **I** (n=3 per experiment), Percentage of IL-17A$^+$ and IFN-γ$^+$ cells in GFP$^-$ or GFP$^+$ cells transduced with LMP empty vector control. Data are representative of two (**b**) to three independent experiments **(a, c–i)** and presented as the mean ± SD **(a,c–i)**. Statistical significance by two-way ANOVA Multiple comparisons. *, $P < 0.05$; **, $P < 0.01$; ***, $P < 0.001$; ****, $P < 0.0001$. Source data and exact *P* value are provided as a Source Data file.

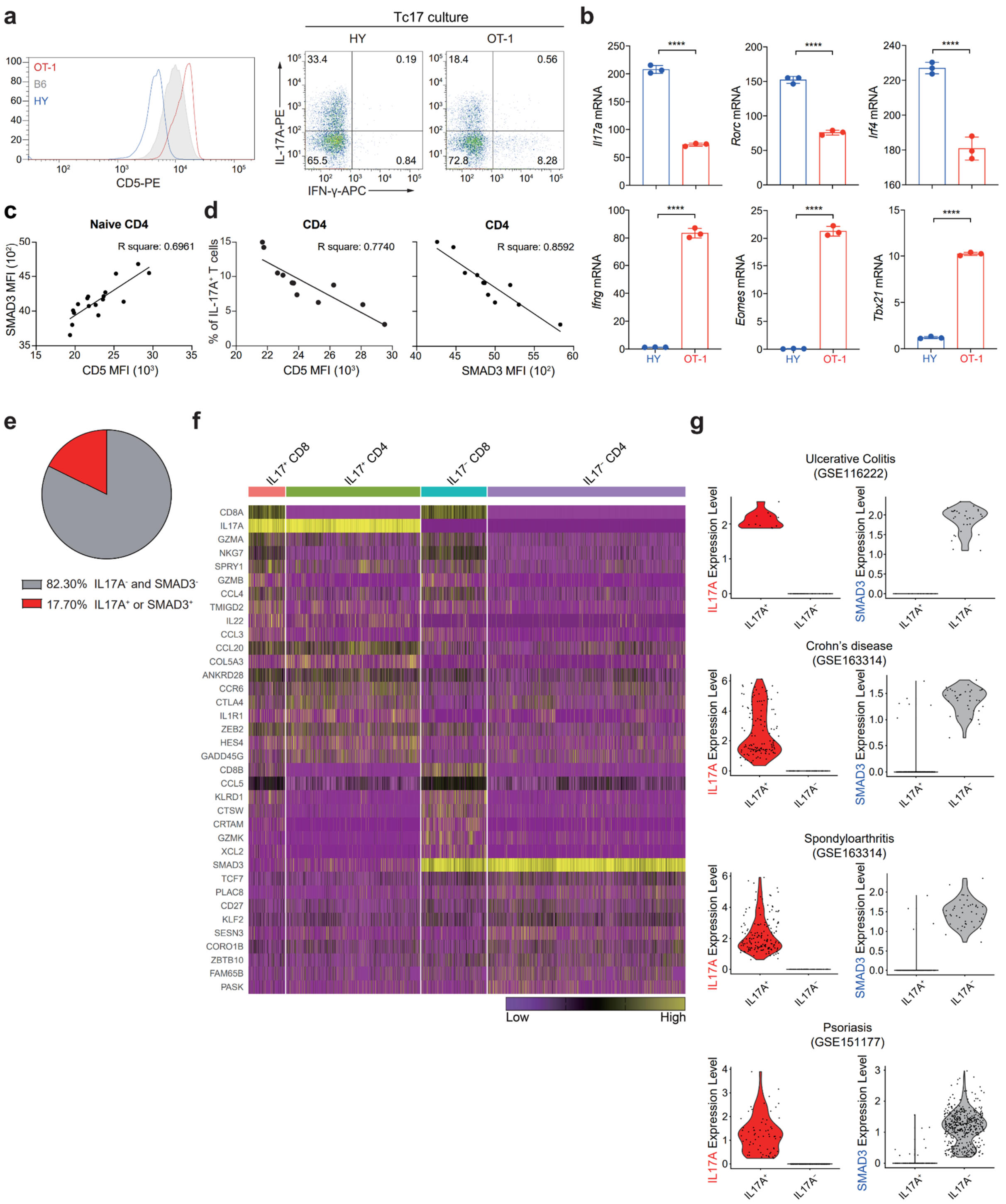

Supplementary Fig. 6

Inverse relationship between CD5 and SMAD3 expression and Tc17 differentiation potential in mice and humans.

a, Expression levels of CD5 shown in histogram for HY and OT-1 *ex vivo* (left), and representative FACS plots for IL-17A/IFN-γ production (right) after Tc17-polarizing culture.

b, qRT-PCR data for *Il17a, Rorc, Irf4, Ifng, Eomes,* and *Tbx21* analyzed for Tc17-polarized HY and OT-1 $CD8^+$ T_N cells **(**n=3 per experiment**)**. **c,** Relationship between CD5 and SMAD3 levels *ex vivo* in $CD4^+$ T_N populations from healthy human bloods **(**n=17**)**. **d,** Relationship between CD5 (left) or SMAD3 (right) levels *ex vivo* and the percentages of IL-$17A^+$ cells after Tc17-polarizing cultures with human $CD4^+$ T_N populations **(**n=11**)**. **e,f,** *IL17A* and *SMAD3* expression profiles in $CD3^+$ cells **(e)** and heatmap of differentially expressed genes of $IL17^+CD8^+$, $IL17^+CD4^+$, $IL\text{-}17^-CD8^+$, and $IL17^-CD4^+$ T cells **(f)** in public scRNA-seq dataset performed with inflammed tissues from UC patients (GSE162335). **g,** *IL17A* and *SMAD3* expression of $CD3^+$ cells that are either $IL17A^+$ or $SMAD3^+$ in public scRNA-seq datasets performed with tissues from patients with Ulcerative Colitis (GSE116222), Crohn's disease (GSE163314), Spondyloarthritis (GSE163314), and Psoriasis (GSE151177), respectively. Data are representative of **(a,b)** or pooled **(c,d)** from two independent experiments and presented as the mean ± SEM **(b–d)**. Statistical significance by simple linear regression two-way ANOVA Multiple comparisons. *, $P < 0.05$; **, $P < 0.01$; ***, $P < 0.001$; ****, $P < 0.0001$. Source data and exact *P* value are provided as a Source Data file.

a

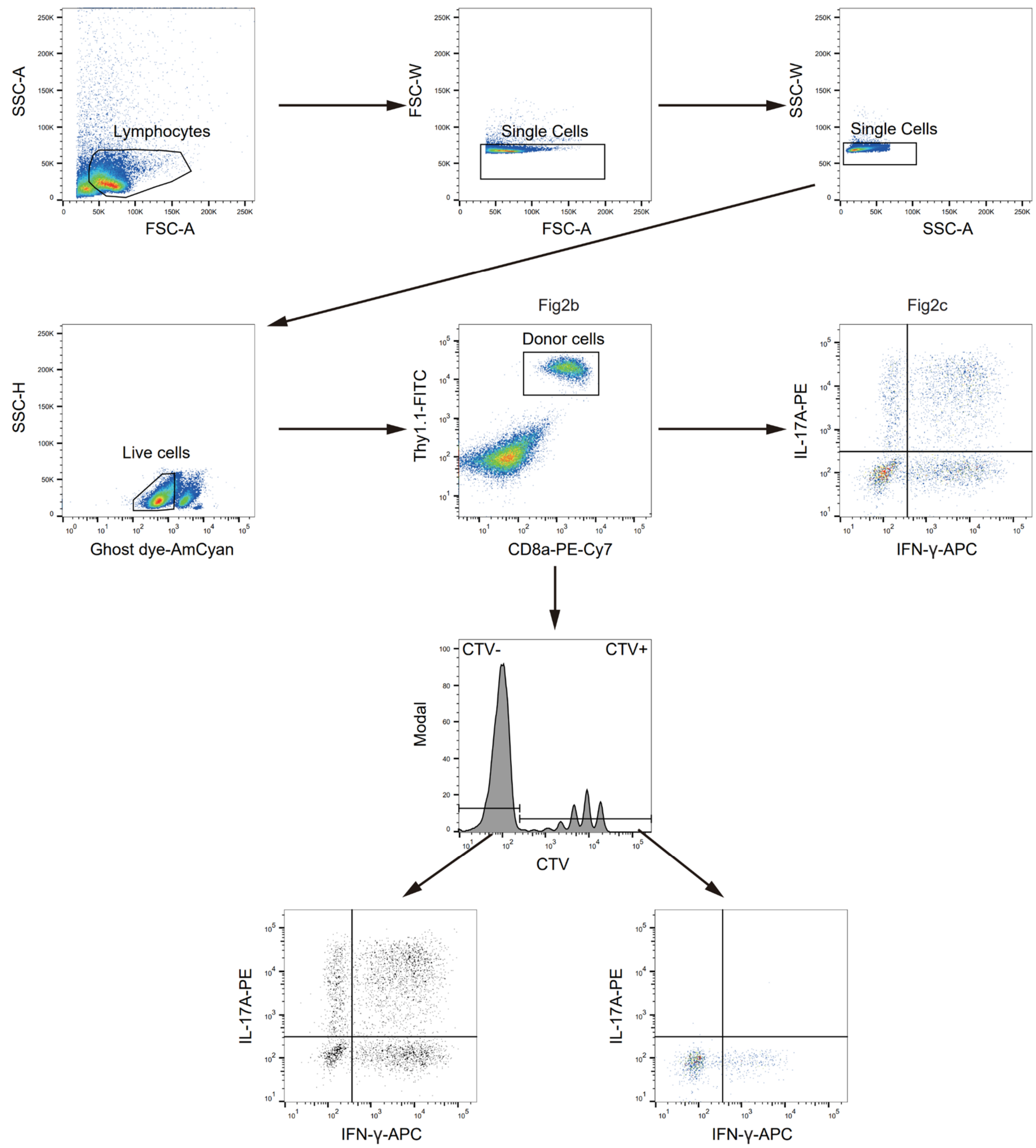

SSC-A
FSC-A
Lymphocytes
FSC-W
Single Cells
SSC-W
SSC-A
Single Cells
SSC-H
Live cells
Ghost dye-AmCyan
Fig2b
Donor cells
Thy1.1-FITC
CD8a-PE-Cy7
Fig2c
IL-17A-PE
IFN-γ-APC
CTV-
CTV+
Modal
CTV
IL-17A-PE
IFN-γ-APC
IL-17A-PE
IFN-γ-APC

Cont.

b

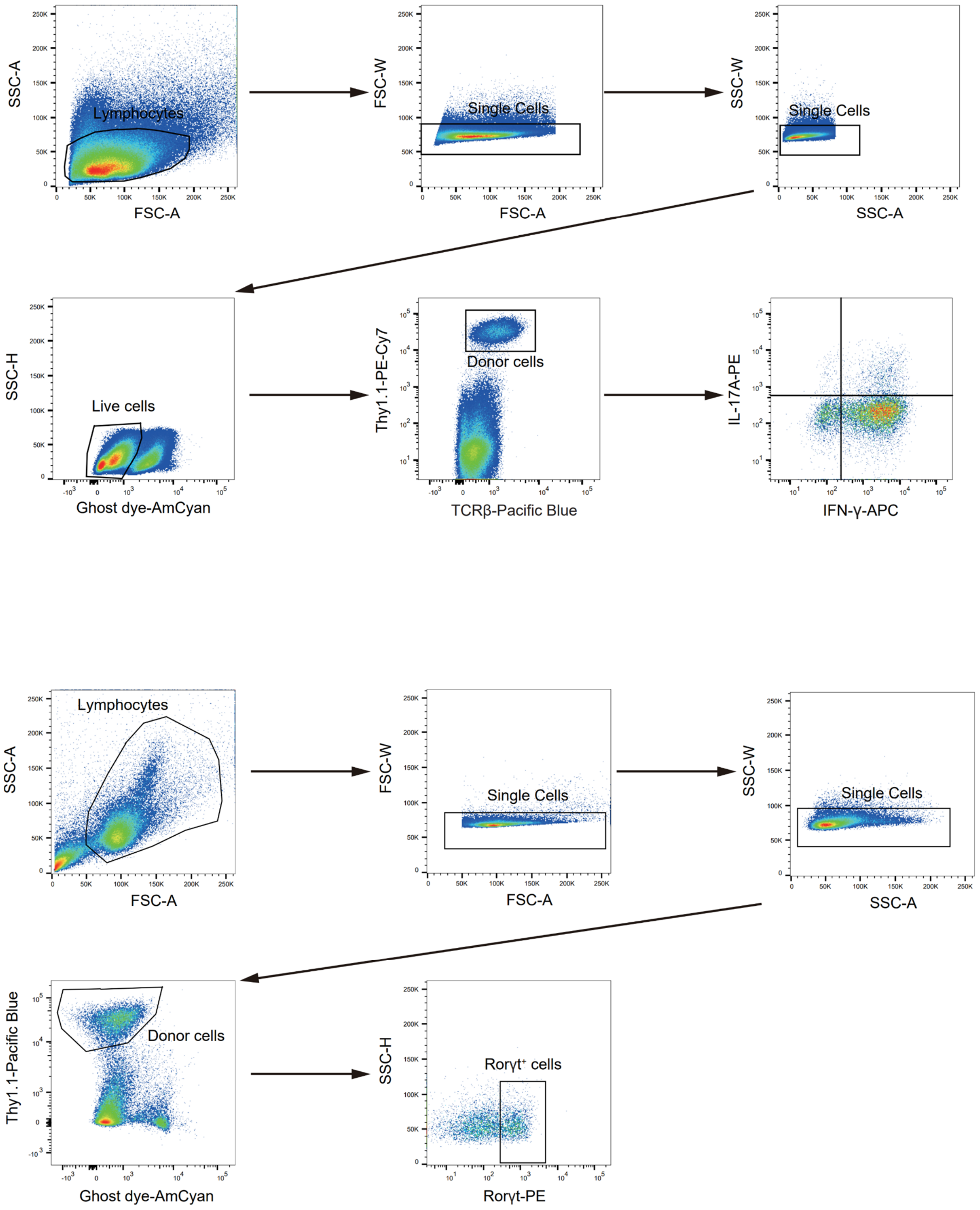
SSC-A
FSC-A
Lymphocytes
FSC-W
FSC-A
Single Cells
SSC-W
SSC-A
Single Cells
SSC-H
Ghost dye-AmCyan
Live cells
Thy1.1-PE-Cy7
TCRβ-Pacific Blue
Donor cells
IL-17A-PE
IFN-γ-APC
SSC-A
FSC-A
Lymphocytes
FSC-W
FSC-A
Single Cells
SSC-W
SSC-A
Single Cells
Thy1.1-Pacific Blue
Ghost dye-AmCyan
Donor cells
SSC-H
Rorγt-PE
Rorγt⁺ cells

Cont.

c

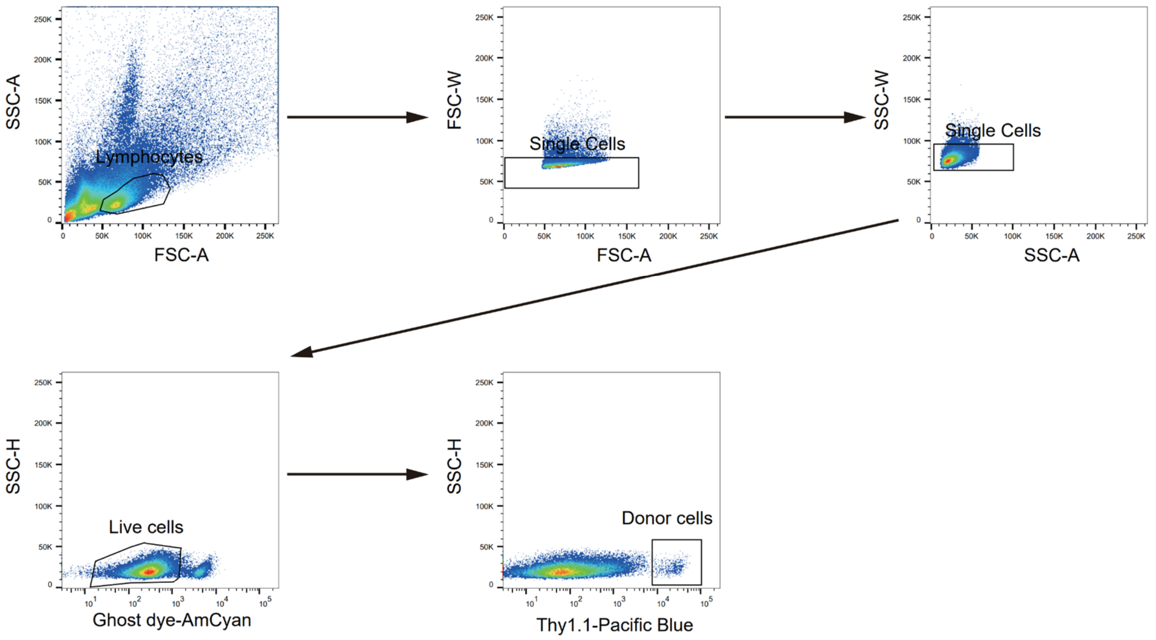
SSC-A
FSC-A
Lymphocytes
FSC-W
Single Cells
SSC-W
SSC-A
Single Cells
SSC-H
Live cells
Ghost dye-AmCyan
SSC-H
Donor cells
Thy1.1-Pacific Blue

d

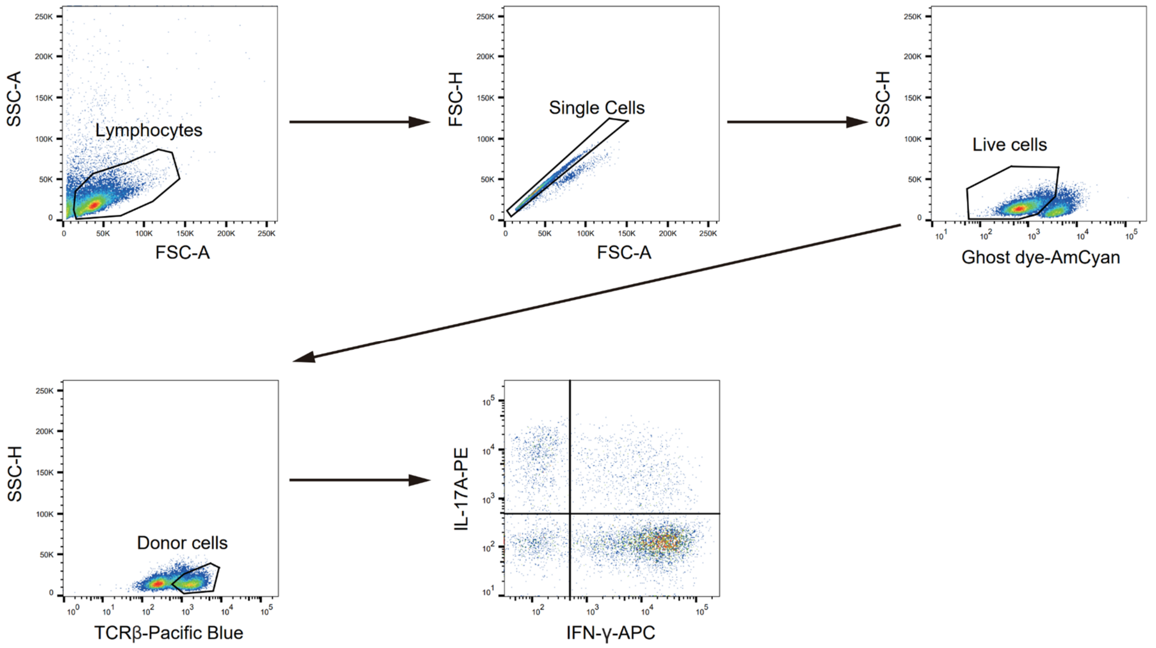
SSC-A
Lymphocytes
FSC-A
FSC-H
Single Cells
FSC-A
SSC-H
Live cells
Ghost dye-AmCyan
SSC-H
Donor cells
TCRβ-Pacific Blue
IL-17A-PE
IFN-γ-APC

Cont.

e

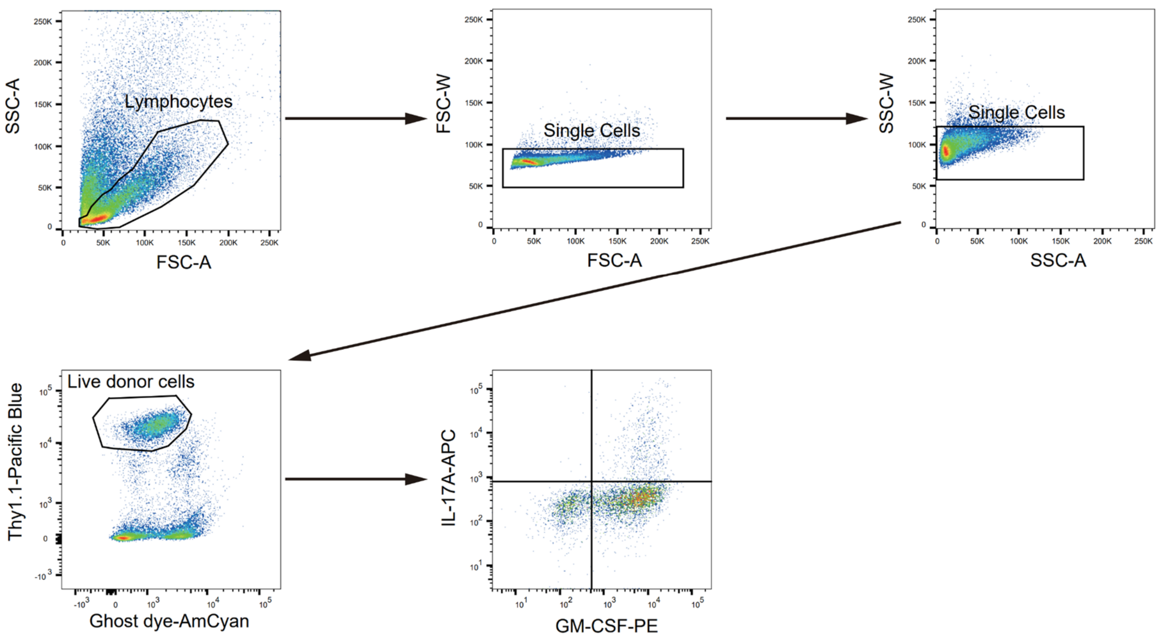
Lymphocytes
SSC-A
FSC-A
Single Cells
FSC-W
FSC-A
Single Cells
SSC-W
SSC-A
Live donor cells
Thy1.1-Pacific Blue
Ghost dye-AmCyan
IL-17A-APC
GM-CSF-PE

f

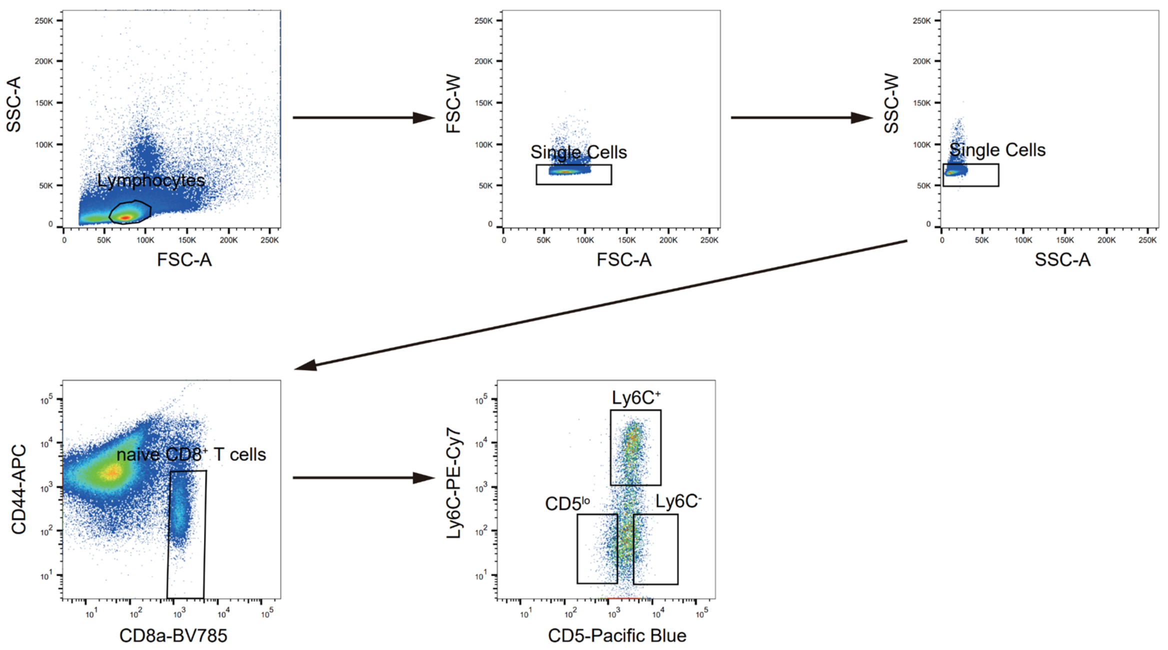
Lymphocytes
SSC-A
FSC-A
Single Cells
FSC-W
FSC-A
Single Cells
SSC-W
SSC-A
naive CD8+ T cells
CD44-APC
CD8a-BV785
Ly6C+
CD5lo
Ly6C-
Ly6C-PE-Cy7
CD5-Pacific Blue

Cont.

g

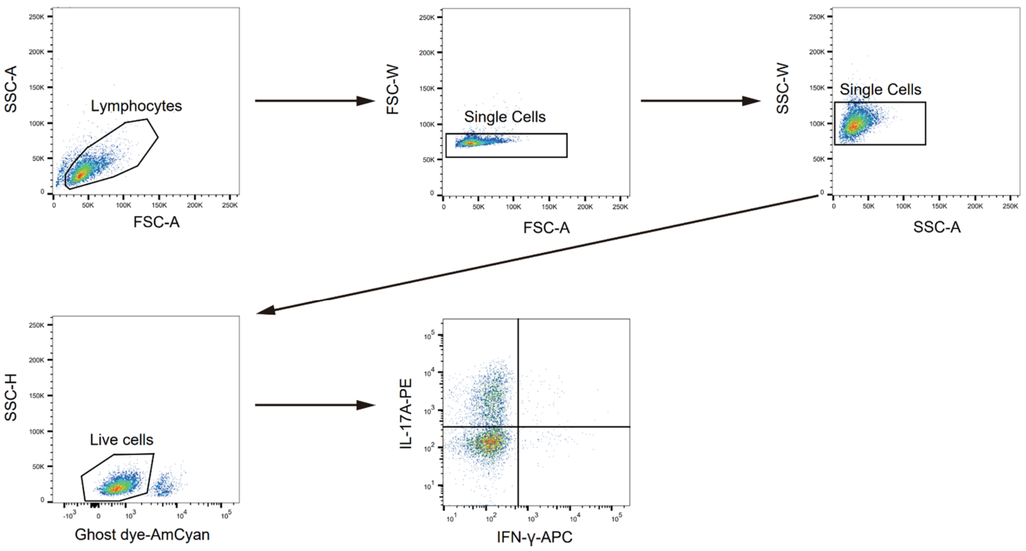
SSC-A
FSC-A
Lymphocytes
FSC-W
Single Cells
SSC-W
SSC-A
SSC-H
Live cells
Ghost dye-AmCyan
IL-17A-PE
IFN-γ-APC

h

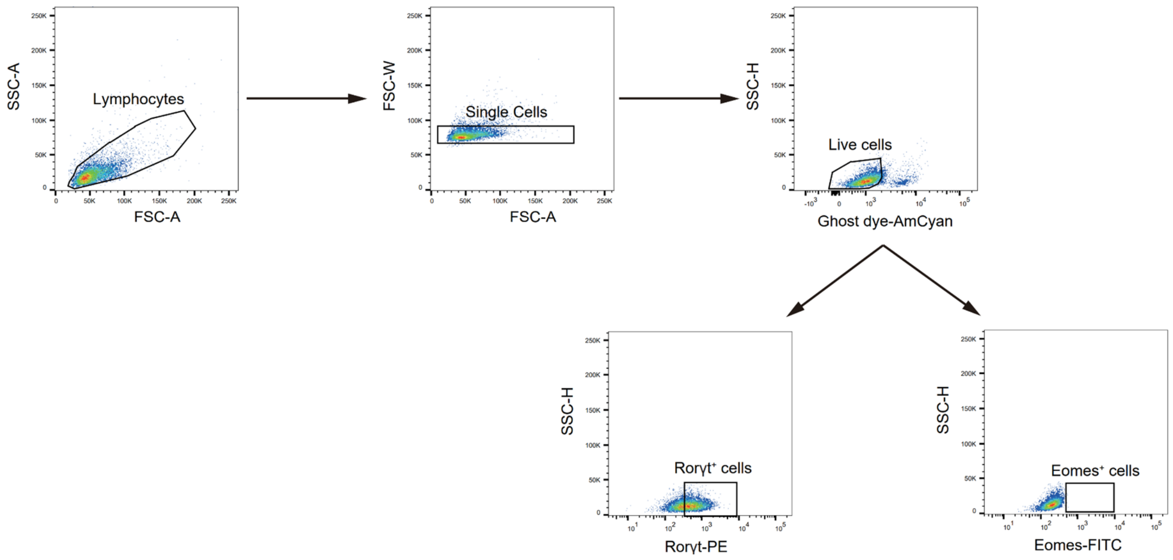
SSC-A
FSC-A
Lymphocytes
FSC-W
Single Cells
SSC-H
Live cells
Ghost dye-AmCyan
Rorγt⁺ cells
Rorγt-PE
Eomes⁺ cells
Eomes-FITC

Cont.

i

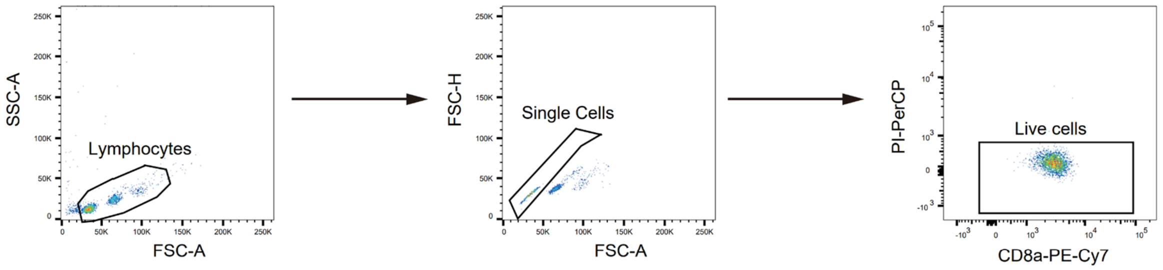

SSC-A
FSC-A
Lymphocytes
FSC-H
Single Cells
PI-PerCP
CD8a-PE-Cy7
Live cells

j

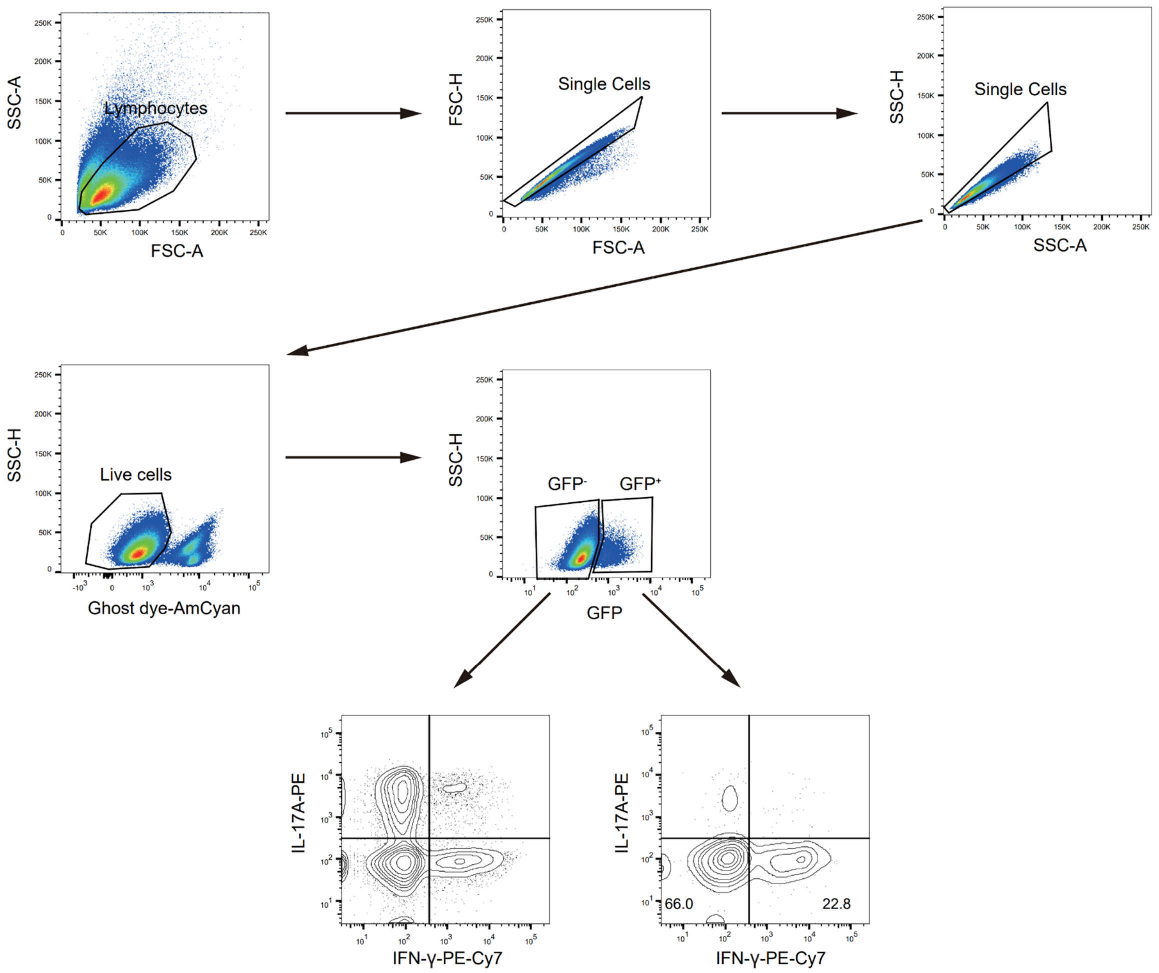

SSC-A
FSC-A
Lymphocytes
FSC-H
Single Cells
SSC-H
SSC-A
Single Cells
SSC-H
Live cells
Ghost dye-AmCyan
SSC-H
GFP-
GFP+
GFP
IL-17A-PE
IFN-γ-PE-Cy7
IL-17A-PE
66.0
22.8
IFN-γ-PE-Cy7

Cont.

k

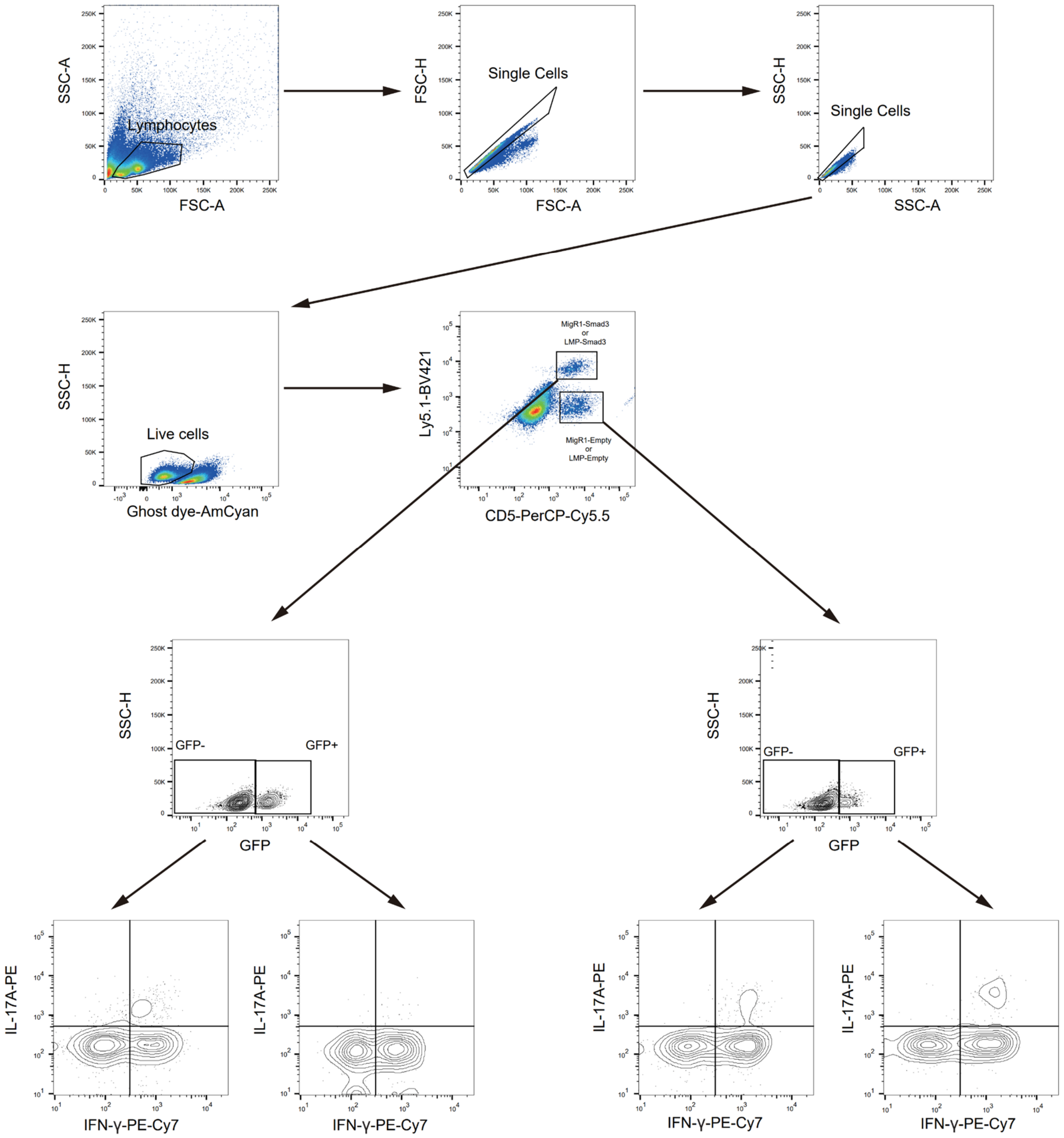
SSC-A
FSC-A
Lymphocytes
FSC-H
Single Cells
SSC-H
SSC-A
Single Cells
Live cells
Ghost dye-AmCyan
Ly5.1-BV421
CD5-PerCP-Cy5.5
MigR1-Smad3
or
LMP-Smad3
MigR1-Empty
or
LMP-Empty
GFP-
GFP+
GFP
IL-17A-PE
IFN-γ-PE-Cy7

Cont.

l

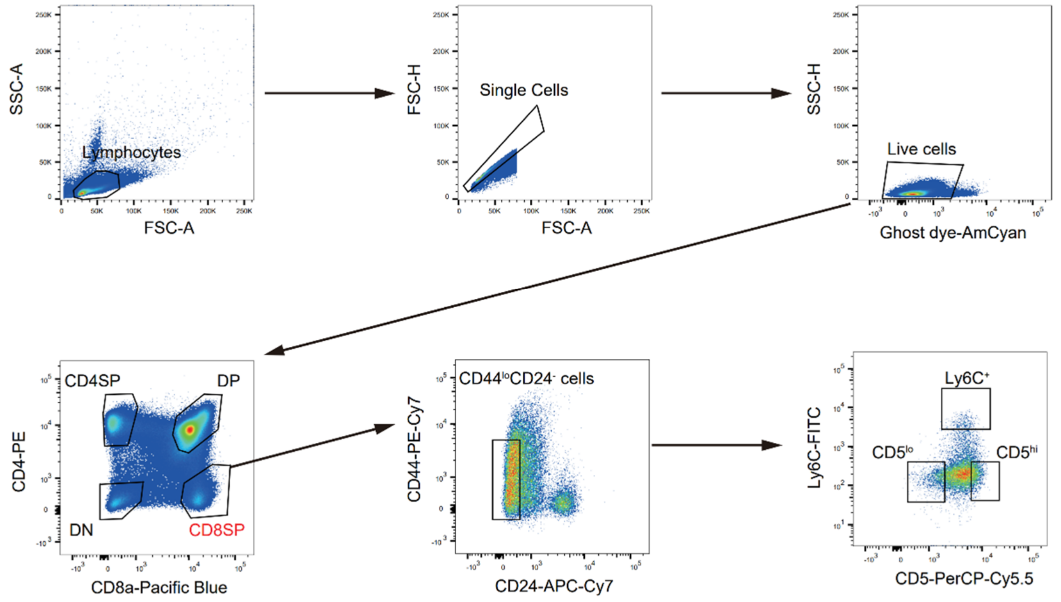
SSC-A
FSC-A
Lymphocytes
FSC-H
Single Cells
SSC-H
Live cells
Ghost dye-AmCyan
CD4-PE
CD4SP
DP
DN
CD8SP
CD8a-Pacific Blue
CD44-PE-Cy7
CD44loCD24- cells
CD24-APC-Cy7
Ly6C-FITC
Ly6C+
CD5lo
CD5hi
CD5-PerCP-Cy5.5

m

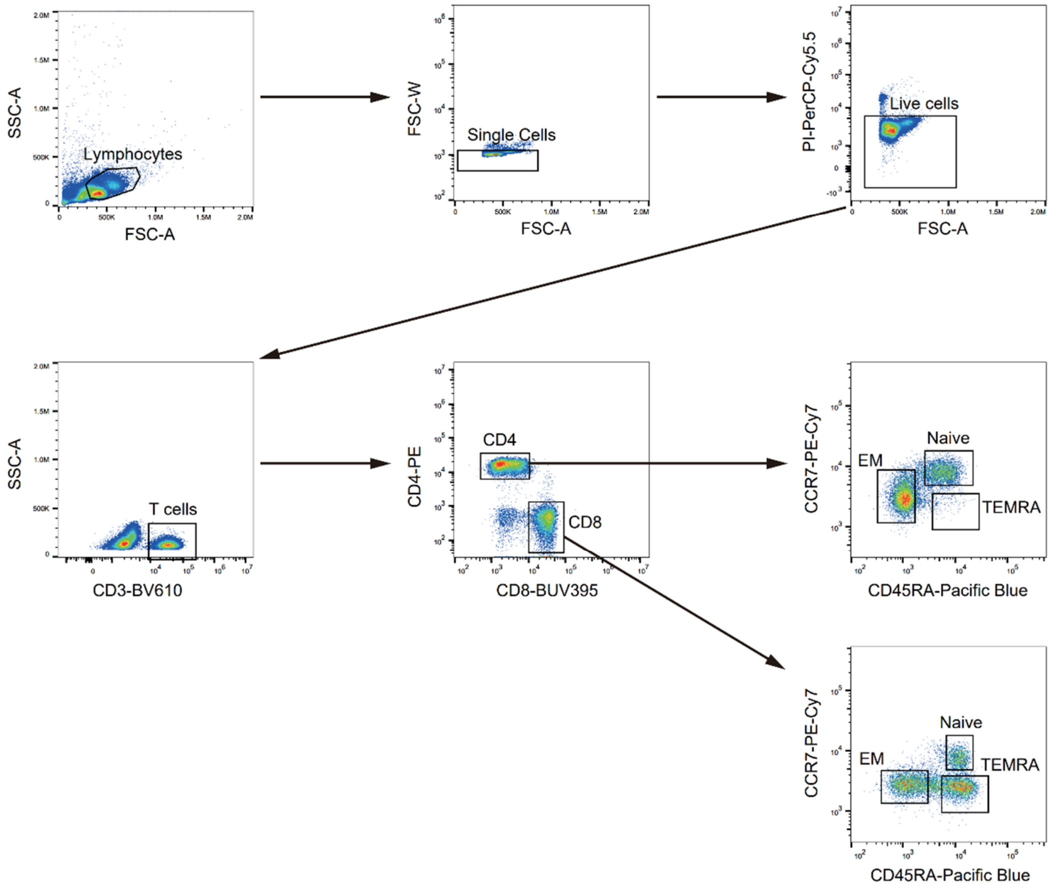
SSC-A
FSC-A
Lymphocytes
FSC-W
Single Cells
PI-PerCP-Cy5.5
Live cells
T cells
CD3-BV610
CD4-PE
CD4
CD8
CD8-BUV395
CCR7-PE-Cy7
Naive
EM
TEMRA
CD45RA-Pacific Blue
CCR7-PE-Cy7
Naive
EM
TEMRA
CD45RA-Pacific Blue

Cont.

n

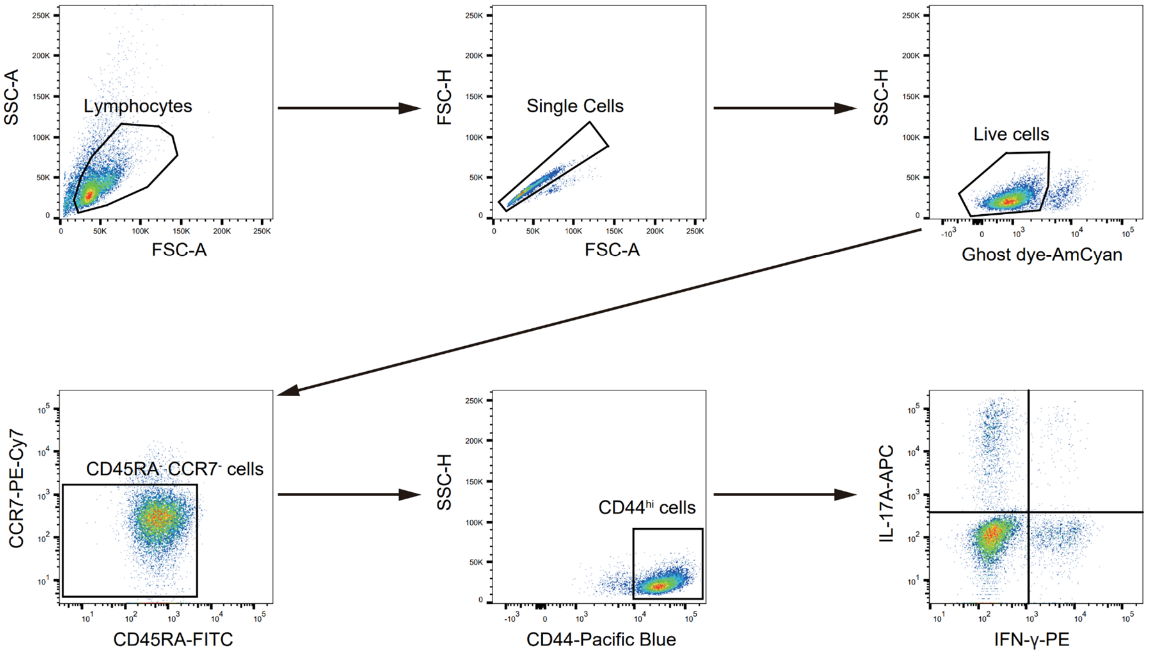

o

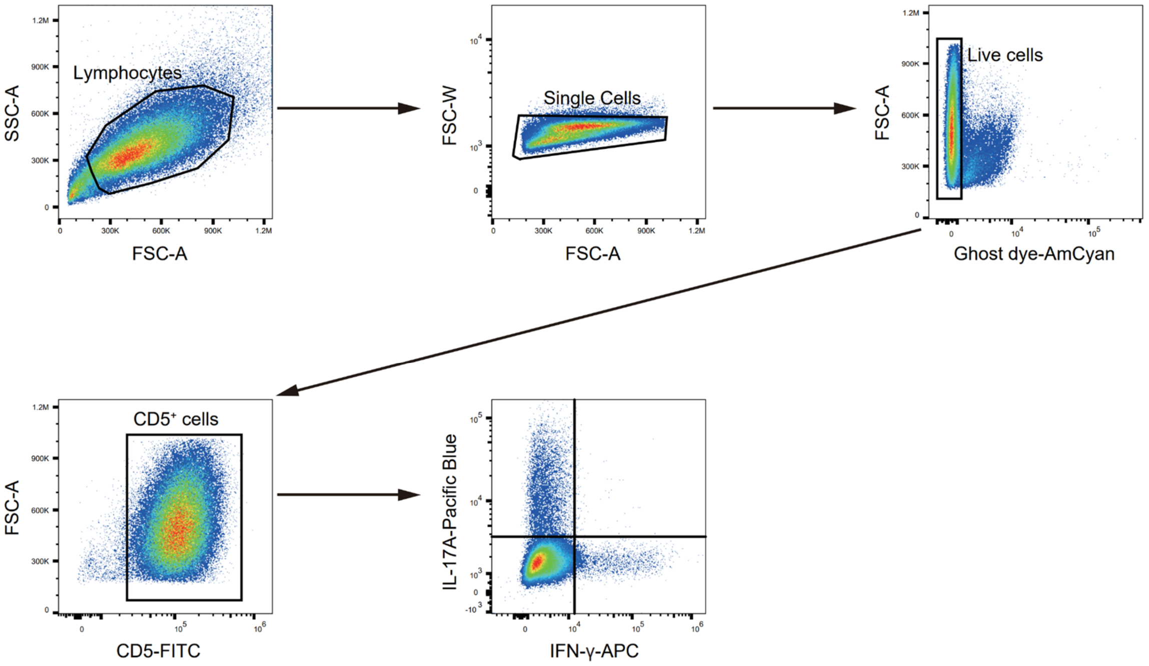

Supplementary Fig. 7

Gating strategies for flow cytometry data.

a, Gating strategy for Fig. 2b,c, and Supplementary Fig. 2a,b. **b,** Gating strategy for Fig. 2e,g,h,i, and Supplementary Fig. 2d–f,g. **c,** Gating strategy for Fig. 3a, and Supplementary Fig. 3a. **d,** Gating strategy for Fig. 3e,g,h, and Supplementary Fig. 3b–l. **e,** Gating strategy for Fig. 4e–h. **f,** Gating strategy for Supplementary Fig4. e. **g,** Gating strategy for Fig. 5b,c,e,g,h,j, Fig. 7c,d, and Supplementary Fig. 4a–c,g–i. **h,** Gating strategy for Fig. 5d. **i,** Gating strategy for Fig. 6a,c, and Supplementary Fig. 5a,d. **j,** Gating strategy for Fig. 6e,f, and Supplementary Fig. 5f–i. **k,** Gating strategy for Fig6. g–j. **l,** Gating strategy for Fig. 7a. **m,** Gating strategy for Fig. 7h, and Supplementary Fig. 6c. **n,** Gating strategy for Fig. 7i, and Supplementary Fig. 6d. **o,** Gating strategy for Fig. 7j.

Antibodies	Manufacturer	Catalog #	Applications	Dilution
anti-CD16/32	ebioscience	14-0161-82	Flow cytometry	1:300
anti-CD3ε (PB); clone 145-2C11	Biolegend	100334	Flow cytometry	1:300
anti-CD5 (PE); clone 53-7.3	Invitrogen	12-0051-83	Flow cytometry	1:300
anti-CD44 (eF450); clone IM7	Invitrogen	48-0441-82	Flow cytometry	1:300
anti-CD62L (PE); clone MEL-14	Biolegend	104408	Flow cytometry	1:300
anti-CD45.1 (BUV395); clone A20	BD Bioscience	565212	Flow cytometry	1:300
anti-CD45.2 (PB); clone 104	Biolegend	109820	Flow cytometry	1:300
anti-CD90.1 (FITC); clone HIS51	Invitrogen	11-0900-85	Flow cytometry	1:300
anti-CD90.2 (PE) (53-2.1)	ebioscience	15298609	Flow cytometry	1:300
anti-Ly6C (PE-cy7) (HK1.4)	ebioscience	15518606	Flow cytometry	1:300
anti-CD8α (APC) (53-6.7)	Tonbo	20-0081-U100	Flow cytometry	1:300
anti-CD126 (APC) (D7715A7)	Biolegend	115812	Flow cytometry	1:300
anti-GP130 (APC); clone KGP130	ThermoFisher	17-1302-82	Flow cytometry	1:300
anti-TGFBRI (APC); clone 141231	R&D biosystems	FAB5871A	Flow cytometry	1:300
anti-TGFBRII (PE); polyclonal	R&D biosystems	FAB532P	Flow cytometry	1:300
anti-α4β7 (APC); clone DATK32	ThermoFisher	17-5887-82	Flow cytometry	1:300
anti-CD195 (PE); clone HM-CCR5	ebioscience	12-1951-81	Flow cytometry	1:300
anti-CD196 (PE); clone 29-2L17	Biolegend	129804	Flow cytometry	1:300
anti-IFN-γ (APC); clone XMG1.2	Invitrogen	17-7311-82	Flow cytometry	1:200
anti-IL-17A (PE-Cy7); clone TC11-18H10.1	Biolegend	506922	Flow cytometry	1:200
anti-IL-17A (PE); clone eBio17B7	Invitrogen	12-7177-81	Flow cytometry	1:200
anti-Eomes (APC)	ebioscience	50-4875-82	Flow cytometry	1:200
anti-Rorgt (PE); clone B2D	Invitrogen	12-6981-82	Flow cytometry	1:200
anti-Rorgt (PE-eF610); clone B2D	Invitrogen	61-6981-82	Flow cytometry	1:200
anti-IRF4 (APC); clone IRF4.3E4	Biolegend	646408	Flow cytometry	1:200
anti-GM-CSF (PE); clone MP1-22E9	Invitrogen	12-7331-82	Flow cytometry	1:200
anti-pERK (PE); clone py204	BD Bioscience	612566	Flow cytometry	1:200
anti-hCD3 (APC-cy7); clone HIT3a	Biolegend	300318	Flow cytometry	1:100
anti-hCD4 (APC); clone A161A1	Biolegend	357408	Flow cytometry	1:100
anti-hCD5 (PE); clone L17F12	Biolegend	364014	Flow cytometry	1:100
anti-hCD8 (FITC); clone SK1	Biolegend	344704	Flow cytometry	1:100
anti-hCD45RA (PB); clone HI100	Biolegend	304123	Flow cytometry	1:100
anti-hCCR7 (PE-cy7); clone G043H7	Biolegend	353225	Flow cytometry	1:100
anti-hIFN-γ (APC); clone B27	Biolegend	506510	Flow cytometry	1:100
anti-hIL-17A (BV421); clone BL168	Biolegend	512322	Flow cytometry	1:100
anti-hIL-17A (PE); clone BL168	Biolegend	512306	Flow cytometry	1:100
anti-pSMAD2; clone 138D4	Cell Signaling Technology	3108	Flow cytometry and Western blot	1:500
anti-pSMAD3; clone C25A9	Cell Signaling Technology	9520	Flow cytometry and Western blot	1:500
anti-IRF4; clone D9P5H	Cell Signaling Technology	15106	Chip assay	1:100
anti-pSTAT3; clone D3A7	Cell Signaling Technology	9145	Western blot	1:1000
anti-Smad2; clone D43B4	Cell Signaling Technology	5339	Flow cytometry	1:500
anti-Smad3; clone C67H9	Cell Signaling Technology	9523	Flow cytometry	1:500
anti-β-actin; clone AC-15	Sigma-Aldrich	A1978	Western blot	1:5000
goat anti-rabbit IgG (AF647)	Invitrogen	A21245	Flow cytometry	1:300
m-IgG_K BP-HRP	Santa Cruz Biotechnology	sc-516102	Western blot	1:1000
goat anti-rabbit IgG-HRP	Santa Cruz Biotechnology	sc-2004	Western blot	1:1000
anti-CD3ε (Purified); clone 145-2C11	ebioscience	16-0031-86	In vitro cell culture	
anti-CD28 (Purified); clone 37.51	ebioscience	16-0281-86	In vitro cell culture	
anti-IFN-γ (Purified); clone XMG1.2	ebioscience	16-7311-85	In vitro cell culture	
anti-IL-4 (Purified); clone 11B11	ebioscience	16-7041-85	In vitro cell culture	
anti-hIFN-γ (Purified); clone B27	Biolegend	506501	In vitro cell culture	
anti-hIL-4 (Purified); clone MP4-25D2	Biolegend	500802	In vitro cell culture	

Supplementary Table. 1

Antibodies.

Primers	Manufacturer	Catalog #
TaqMan probe_*Rorc*	Applied Biosystems	Mm01261022_m1
TaqMan probe_*Il17a*	Applied Biosystems	Mm00439618_m1
TaqMan probe_*Irf4*	Applied Biosystems	Mm00516431_m1
TaqMan probe_*Ifnγ*	Applied Biosystems	Mm01168134_m1
TaqMan probe_*Tbx21*	Applied Biosystems	Mm00450960_m1
TaqMan probe_*Eomes*	Applied Biosystems	Mm01351984_m1
TaqMan probe_*Rn18sRn45s*	Applied Biosystems	Mm03928990_g1

Rorc region (IRF4)	Sense primer	Antisense primer
N.C_-1576 ~ -1737	TGAGCACACTATCACTCTCTTCAG	TGACCCTTGGGTAGGAGAGA
Target_+10747 ~ +10824	GGGCCCTGAGATGGTAAGTT	GGGTGCTGAGTAATCACAGGA

Il17a region (IRF4)	Sense primer	Antisense primer
N.C_-3387 ~ -3523	CTCCCATGTGGTCATTATTGC	GTGTCCTTAGGTCCTAAATGTAGG
Target_-1592~-1815	AATCCATGGAGCTGGAGAGA	TTTTTATACAACATAGGTCTTCATGG

Rorc region (Smad3)	Sense primer	Antisense primer
N.C_-727 ~ -881	GGTTGTTGGGTAAGCAGGAA	CACGACCCCGTAATTCTGTT
Target_-862 ~ -1181	CAACGGTGGAGAATGGAATG	TTCCTGCTTACCCAACAACC

Il17a region (Smad3)	Sense primer	Antisense primer
N.C_-1211 ~ -984	CAGGGATAATGCCAAGGGTA	AGCATGAGGTGGACCGATAG
Target_-11~ -185	AACTTCTGCCCTTCCCATCT	GCTCCTTTCTCTCTTTTTATACGG

Smad3 overexpression	
Forward	GACTCGAGATGTCGTCCATCCTGCCC
Reverse	GAGAATTCCTAAGACACACTTTAACAGCG

shRNA sequence for Smad3	TGCTGTTGACAGTGAGCGAACGCAGAACGTGAACACCAAGTAG TGAAGCCACAGATGTACTTGGTGTTCACGTTCTGCGTGTGCCTAC

Supplementary Table. 2

Primers.
