# Supplementary material for: Developmental self-reactivity determines pathogenic Tc17 differentiation potential of naive CD8+ T cells in murine models of inflammation
Source: Nat Commun. 2024 Apr 4;15:2919. doi: 10.1038/s41467-024-47144-4 (PMC10994929; doi:10.1038/s41467-024-47144-4)
Supplement: Supplementary file 3 — Reporting Summary [file 41467_2024_47144_MOESM3_ESM.pdf]

nature portfolio

Corresponding author(s): Jae Ho Cho
Last updated by author(s): Jan 24, 2024

Reporting Summary

Nature Portfolio wishes to improve the reproducibility of the work that we publish. This form provides structure for consistency and transparency in reporting. For further information on Nature Portfolio policies, see our Editorial Policies and the Editorial Policy Checklist.

Statistics

For all statistical analyses, confirm that the following items are present in the figure legend, table legend, main text, or Methods section.

n/a	Confirmed	
☐	☒	The exact sample size (*n*) for each experimental group/condition, given as a discrete number and unit of measurement
☐	☒	A statement on whether measurements were taken from distinct samples or whether the same sample was measured repeatedly
☐	☒	The statistical test(s) used AND whether they are one- or two-sided<br>*Only common tests should be described solely by name; describe more complex techniques in the Methods section.*
☒	☐	A description of all covariates tested
☒	☐	A description of any assumptions or corrections, such as tests of normality and adjustment for multiple comparisons
☐	☒	A full description of the statistical parameters including central tendency (e.g. means) or other basic estimates (e.g. regression coefficient) AND variation (e.g. standard deviation) or associated estimates of uncertainty (e.g. confidence intervals)
☐	☒	For null hypothesis testing, the test statistic (e.g. *F*, *t*, *r*) with confidence intervals, effect sizes, degrees of freedom and *P* value noted<br>*Give P values as exact values whenever suitable.*
☒	☐	For Bayesian analysis, information on the choice of priors and Markov chain Monte Carlo settings
☒	☐	For hierarchical and complex designs, identification of the appropriate level for tests and full reporting of outcomes
☒	☐	Estimates of effect sizes (e.g. Cohen's *d*, Pearson's *r*), indicating how they were calculated

Our web collection on statistics for biologists contains articles on many of the points above.

Software and code

Policy information about availability of computer code

Data collection	Flow cytometry - BD Bioscience FACSDiva 7.0<br>Quantitative Real-time PCR - StepOne Software 2.2.2<br>Immunofluorescence image - Leica TCS SP5 - Leica Application Suite X version 3.7.4.23463
Data analysis	TreeStar FlowJo 10.6.2<br>GraphPad Prism 8.4.1<br>Broad Institute Gene Set Enrichment Analysis version 4.0.1<br>Single cell RNA sequencing data - Seurat (R) version 4.3.0<br>ImageJ version 1.5.3

For manuscripts utilizing custom algorithms or software that are central to the research but not yet described in published literature, software must be made available to editors and reviewers. We strongly encourage code deposition in a community repository (e.g. GitHub). See the Nature Portfolio guidelines for submitting code & software for further information.

Data

Policy information about availability of data

All manuscripts must include a data availability statement. This statement should provide the following information, where applicable:

- Accession codes, unique identifiers, or web links for publicly available datasets
- A description of any restrictions on data availability
- For clinical datasets or third party data, please ensure that the statement adheres to our policy

Bulk RNA-Seq data has been deposited in the NCBI's Gene Expression Omnibus (GEO) database under the primary accession number GSE240440 [https://www.ncbi.nlm.nih.gov/geo/query/acc.cgi?acc=GSE240440]. The datasets generated during the current study are available from the corresponding author (JHC) upon request. Following public scRNA-seq datasets can be accessed with the links provided below:
GSE162335 [https://www.ncbi.nlm.nih.gov/geo/query/acc.cgi?acc=GSE162335], GSE163314 [https://www.ncbi.nlm.nih.gov/geo/query/acc.cgi?acc=GSE163314], GSE151177 [https://www.ncbi.nlm.nih.gov/geo/query/acc.cgi?acc=GSE151177], GSE116222 [https://www.ncbi.nlm.nih.gov/geo/query/acc.cgi?acc=GSE116222].
The authors declare that all data supporting the findings of this study are available within the paper. Source data are provided with this paper.

Research involving human participants, their data, or biological material

Policy information about studies with human participants or human data. See also policy information about sex, gender (identity/presentation), and sexual orientation and race, ethnicity and racism.

Reporting on sex and gender	For human blood samples included in this study, all the cases were de-identified. No sex and gender specific analysis was carried out.
Reporting on race, ethnicity, or other socially relevant groupings	Not specified.
Population characteristics	All samples were taken from healthy participants. Sex, gender, and age were not considered as biological variables in this study.
Recruitment	Healthy participants were recruited at the Korean Red Cross.
Ethics oversight	All samples were collected and used in accordance with a protocol approved by the Chonnam National University Hwasun Hospital (CNUHH) ethics committee (CNUHH-2021-045). A written informed consent exemption was obtained from the Institutional Review Boards for the blood used in this research because it was impossible to obtain written consent from research subjects, there was no reason to presume refusal of consent from research subjects, and the risk to research subjects was extremely low.

Note that full information on the approval of the study protocol must also be provided in the manuscript.

Field-specific reporting

Please select the one below that is the best fit for your research. If you are not sure, read the appropriate sections before making your selection.

☒ Life sciences ☐ Behavioural & social sciences ☐ Ecological, evolutionary & environmental sciences

For a reference copy of the document with all sections, see nature.com/documents/nr-reporting-summary-flat.pdf

Life sciences study design

All studies must disclose on these points even when the disclosure is negative.

Sample size	Group sizes were determined on the basis of preliminary experiments and were found sufficient to reveal biologically relevant differences among the samples of interest.
Data exclusions	No data were excluded from the analyses.
Replication	All experiments were repeated at least twice and and gave comparable results each time.
Randomization	Mice were allocated to experimental groups based on sex and age matched within experiments.
Blinding	Investigators were not blinded to group allocation. Since most experiments were done by one investigator, the investigator needed to know the exact condition of the experimenting mice. To avoid bias of the investigator, all mice were bred and experimented on the same day with the same procedures, analysis were carried out with authorized softwares using strict standards (e.g. gating strategy). Therefore, no blinding was necessary.

Reporting for specific materials, systems and methods

We require information from authors about some types of materials, experimental systems and methods used in many studies. Here, indicate whether each material, system or method listed is relevant to your study. If you are not sure if a list item applies to your research, read the appropriate section before selecting a response.

Materials & experimental systems

n/a	Involved in the study
☐	☒ Antibodies
☐	☒ Eukaryotic cell lines
☒	☐ Palaeontology and archaeology
☐	☒ Animals and other organisms
☒	☐ Clinical data
☒	☐ Dual use research of concern
☒	☐ Plants

Methods

n/a	Involved in the study
☒	☐ ChIP-seq
☐	☒ Flow cytometry
☒	☐ MRI-based neuroimaging

Antibodies

Antibodies used

Antibody, clone, Manufacturer, Cat#

anti-CD16/32 ebioscience Cat#: 14-0161-82
anti-CD3ε (PB); clone 145-2C11 Biolegend Cat#: 100334
anti-CD5 (PE); clone 53-7.3 Invitrogen Cat#: 12-0051-83
anti-CD44 (eF450); clone IM7 Invitrogen Cat#: 48-0441-82
anti-CD62L (PE); clone MEL-14 Biolegend Cat#: 104408
anti-CD45.1 (BUV395); clone A20 BD Bioscience Cat#: 565212
anti-CD45.2 (PB); clone 104 Biolegend Cat#: 109820
anti-CD90.1 (FITC); clone HIS51 Invitrogen Cat#: 11-0900-85
anti-CD90.2 (PE) (53-2.1) ebioscience Cat#: 15298609
anti-Ly6C (PE-cy7) (HK1.4) ebioscience Cat#: 15518606
anti-CD8α (APC) (53-6.7) Tonbo Cat#: 20-0081-U100
anti-CD126 (APC) (D7715A7) Biolegend Cat#: 115812
anti-GP130 (APC); clone KGP130 ThermoFisher Cat#: 17-1302-82
anti-TGFBRI (APC); clone 141231 R&D biosystems Cat#: FAB5871A
anti-TGFBRII (PE); polyclonal R&D biosystems Cat#: FAB532P
anti-α4β7 (APC); clone DATK32 ThermoFisher Cat#: 17-5887-82
anti-CD195 (PE); clone HM-CCR5 ebioscience Cat#:12-1951-81
anti-CD196 (PE); clone 29-2L17 Biolegend Cat#: 129804
anti-IFN-γ (APC); clone XMG1.2 Invitrogen Cat#: 17-7311-82
anti-IL-17A (PE-cy7); clone TC11-18H10.1 Biolegend Cat#: 506922
anti-IL-17A (PE); clone eBio17B7 Invitrogen Cat#: 12-7177-81
anti-Eomes (APC) ebioscience Cat#: 50-4875-82
anti-Rorgt (PE); clone B2D Invitrogen Cat#: 12-6981-82
anti-Rorgt (PE-eF610); clone B2D Invitrogen Cat#: 61-6981-82
anti-IRF4 (APC); clone IRF4.3E4 Biolegend Cat#: 646408
anti-GM-CSF (PE); clone MP1-22E9 Invitrogen Cat#: 12-7331-82
anti-pERK (PE); clone py204 BD Bioscience Cat#: 612566
anti-hCD3 (APC-cy7); clone HIT3a Biolegend Cat#: 300318
anti-hCD4 (APC); clone A161A1 Biolegend Cat#: 357408
anti-hCD5 (PE); clone L17F12 Biolegend Cat#: 364014
anti-hCD8 (FITC); clone SK1 Biolegend Cat#: 344704
anti-hCD45RA (PB); clone HI100 Biolegend Cat#: 304123
anti-hCCR7 (PE-cy7); clone G043H7 Biolegend Cat#: 353225
anti-hIFN-γ (APC); clone B27 Biolegend Cat#: 506510
anti-hIL-17A (BV421); clone BL168 Biolegend Cat#: 512322
anti-hIL-17A (PE); clone BL168 Biolegend Cat#: 512306
anti-pSMAD2; clone 138D4 Cell Signaling Technology Cat#: 3108
anti-pSMAD3; clone C25A9 Cell Signaling Technology Cat#: 9520
anti-pSTAT3; clone D3A7 Cell Signaling Technology Cat#: 9145
anti-IRF4; clone D9P5H Cell Signaling Technology Cat#: 15106
anti-Smad2; clone D43B4 Cell Signaling Technology Cat#: 5339
anti-Smad3; clone C67H9 Cell Signaling Technology Cat#: 9523
anti-β-actin; clone AC-15 Sigma-Aldrich Cat#: A1978
goat anti-rabbit IgG (AF647) Invitrogen Cat#: A21245
m-IgGK BP-HRP Santa Cruz Biotechnology Cat#: sc-516102
goat anti-rabbit IgG-HRP Santa Cruz Biotechnology Cat#: sc-2004
anti-CD3ε (Purified); clone 145-2C11 ebioscience Cat#: 16-0031-86
anti-CD28 (Purified); clone 37.51 ebioscience Cat#: 16-0281-86

	anti-IFN-γ (Purified); clone XMG1.2 ebioscience Cat#: 16-7311-85<br>anti-IL-4 (Purified); clone 11B11 ebioscience Cat#: 16-7041-85<br>anti-hIFN-γ (Purified); clone B27 Biolegend Cat#: 506501<br>anti-hIL-4 (Purified); clone MP4-25D2 Biolegend Cat#: 500802
Validation	All antibodies used are commercially available, and were validated with mouse for western blot(anti-phospho-STAT3, anti-phospho-Smad2, anti-phospho-Smad3, anti-phospho-ERK1/2 anti-b-actin, goat anti-rabbit IgG-HRP) and flow cytometry (all the antibodies except for those used for western blot) application by the manufactures. Validation statement for each antibody is provided on manufacture's websites.

Eukaryotic cell lines

Policy information about cell lines and Sex and Gender in Research

Cell line source(s)	Platinum-E retroviral packaging cell line was bought from Cell Biolabs Inc. (Cat# RV-101).
Authentication	No specific authentication of the cell lines was performed.
Mycoplasma contamination	This cell line is negative for mycoplasma contamination.
Commonly misidentified lines<br>(See ICLAC register)	No commonly misidentified cell lines were used in the study.

Animals and other research organisms

Policy information about studies involving animals; ARRIVE guidelines recommended for reporting animal research, and Sex and Gender in Research

Laboratory animals	C57BL/6 (B6), and BALB/c mice were purchased from The Jackson Laboratory. B6.SJL (Ly5.1), B6.PL (Thy1.1), P14, OT-I, HY, and Rag1-/- mice (all on a B6 background) were kindly provided by Dr. Kwang Soon Kim (Pohang University of Science and Technology, Pohang, Korea), and Ccr6-/- mice were kindly provided by Dr. You-Me Kim (Korea Advanced Institute of Science and Technology, Daejeon, Korea). All mice were maintained under specific pathogen-free conditions at the Laboratory Animal Center of Chonnam National University (CNU) Medical School. The mouse housing conditions were maintained at 21-23 ℃ room temperature (RT), 50-70% relative humidity, and 12 h light/dark cycle. Both male and female mice, unless described otherwise, at 6 to 8 weeks of age were used in the experiments. Sex was not considered as a biological variable, as no differences related to the overall immune contexts were observed between the male and female mice.
Wild animals	No wild animals were used in this study.
Reporting on sex	Age-matched male or female mice (in all strains) were used.
Field-collected samples	This study did not involve these samples.
Ethics oversight	All animal experiments were performed in accordance with protocols approved by the Animal Experimental and Ethics Committee at Chonnam National University (ethics approval no. CNU IACUC-H-2022-16).

Note that full information on the approval of the study protocol must also be provided in the manuscript.

Plants

Seed stocks	N/A
Novel plant genotypes	N/A
Authentication	N/A

Flow Cytometry

Plots

Confirm that:

☒ The axis labels state the marker and fluorochrome used (e.g. CD4-FITC).

☒ The axis scales are clearly visible. Include numbers along axes only for bottom left plot of group (a 'group' is an analysis of identical markers).

☒ All plots are contour plots with outliers or pseudocolor plots.

☒ A numerical value for number of cells or percentage (with statistics) is provided.

Methodology

Sample preparation	Isolated mouse LN and spleen suspensions were prepared by gently pressing LN and spleen trough 70µm cell strainer and washed with media. In case of spleen, to remove red blood cells, the suspension was resuspended with RBC lysis buffer for 3 min on ice, and wash with media again. The isolated single cells were prepared and stained with flourochrome-conjugated antibodies appropriating to each experimental design indicated on method section and legends.<br>The isolated colon was cut vertically and washed three times with cold PBS. The intestines were then cut into 0.5 mm pieces and washed with 0.5 M EDTA and 3% FBS containing PBS for 20 min using a magnetic stirrer in an incubator at 37°C. Intestinal tissues were washed with cold PBS by shaking vigorously three times. Washed intestinal tissue was digested with collagenase type 4 and DNase I for 40 min using a magnetic stirrer in a 37°C incubator. Enzyme digested suspensions were filtered trough 70µm cell strainer and immune cells were enriched by Percall gradient centrifugation. The isolated single cells were prepared and stained with flourochrome-conjugated antibodies appropriating to each experimental design indicated on method section and legends.
Instrument	Samples were analyzed by FACS Canto II and LSR II (BD). Cell sorting was performed by utilizing MoFlo Astrios or XDP (Beckman coulter)
Software	Data was collected utilizing FACSDiva software (BD) and analyzed by FlowJo (TreeStar). Statistics of data were conducted by Prism (GraphPad).
Cell population abundance	Cell subsets were sorted with >98% purity as controlled by remeasurement of sorted populations.
Gating strategy	Lympocytes were initially gated by FSC(A)/SSC(A), followed by FSC(W/H) to exclude cell doublets. Also dead cells were gated out by utilizing with viability dye before further gating for analyses. Further detail gating strategies were indicated on each figure and legend.

☒ Tick this box to confirm that a figure exemplifying the gating strategy is provided in the Supplementary Information.
